# Assessment of walking disorder in community-dwelling Japanese middle-aged and elderly women using an inertial sensor

**DOI:** 10.7717/peerj.11269

**Published:** 2021-04-14

**Authors:** Toshinori Miyashita, Shintarou Kudo, Yoshihiro Maekawa

**Affiliations:** 1 Graduate School of Health Sciences, Morinomiya University of Medical Sciences, Osaka City, Japan; 2 Department of Physical Therapy, Morinomiya University of Medical Sciences, Osaka City, Japan; 3 Department of Clinical Laboratory, Morinomiya University of Medical Sciences, Osaka City, Japan; 4 Inclusive Medical Science Research Institute, Morinomiya University of Medical Sciences, Osaka City, Japan

**Keywords:** Inertial sensor, Ankle plantar flexor power, Walking speed, Middle-aged, Elderly, Walking assessment

## Abstract

**Background:**

Decreased walking speed has been revealed to be related to many negative events. Several researchers support the importance of triceps surae function as a cause of decreased walking speed. The purpose of this study was to investigate the relationship between walking speed and plantar flexor power during the terminal stance of gait in community-dwelling middle-aged and elderly women using an inertial sensor.

**Methods:**

One hundred thirty-six healthy female middle-aged to elderly community-dwelling women were included in this study. We measured two-step score, grip strength, walking speed and accelerometer data from which we estimated ankle power (estimated ankle power) during walking using an inertial sensor. All participants were classified into the four different age strata, fifties (50–59), sixties (60–69), seventies (70–79) and eighties (80–89). The differences in each parameter between the four age groups were compared using repeated analysis of variance and post-hoc Bonferroni corrections for multiple comparisons to establish significance. Multiple regression analysis was carried out using a stepwise method to determine the correlations with comfortable walking speed. Comfortable walking speed was considered a dependent variable.

**Results:**

The normalized estimated ankle power of the eighties group was significantly decreased in comparison with seventies age groups and fifties age groups (*P* < 0.05), but there were no significant differences in normalized estimated ankle power between the sixties and eighties age-groups. The results of stepwise multiple regression analysis revealed that the normalized estimated ankle power, two-step value and body weight were highly-significant partial regression coefficients (adjusted *R*^2^ = 0.57).

## Introduction

Walking ability is a common but important factor affecting life-space mobility (May, Nayak & Isaacs, 1985), promoting physical activity (Tudor-Locke et al., 2011; Päivi, Mirja & Terttu, 2010; Simpson et al., 2003) and activities of daily of living (ADL) (Perry et al., 1995; Viosca et al., 2005; Fulk et al., 2017), and improving or maintaining quality of life (Rantakokko et al., 2013; Schmid et al., 2007) in community-dwelling people. Previous studies have classified walking ability by walking speed (Perry et al., 1995; Viosca et al., 2005). The usefulness of measuring walking speed is high (Fritz & Lusardi, 2009; Cummings, Studenski & Ferrucci, 2014), and it is used in many studies (Sustakoski et al., 2015; Yoshimura et al., 2015; Graf et al., 2005; Bohannon, 1997; Bohannon & Williams Andrews, 2011; Verlinden et al., 2013; Hollman, McDade & Petersen, 2011).

Decreased walking speed has been revealed to be related to negative events such as decreased survival (Studenski et al., 2011), increased fall risk (Menz, Lord & Fitzpatrick, 2003a, 2003b) and a decline in the ability to perform ADL and maintain life-space mobility (Perry et al., 1995; Viosca et al., 2005; Fulk et al., 2017). Consequently it is important to prevent a decline in walking ability such as decreased walking speed or walking stability.

Several researchers support the importance of triceps surae function as a cause of decreased walking speed (Sutherland, Cooper & Daniel, 1980; Meinders, Gitter & Czerniecki, 1998; Neptune, Kautz & Zajac, 2001; Neptune & McGowan, 2011). Other researchers have reported kinetic data indicating that push-off power, peak plantar flexor moment and plantar flexor power during the terminal stance phase are decreased in elderly walkers compared with those of young persons (Winter et al., 1990; Judge, Ounpuu & Davis, 1996; Franz & Kram, 2013; Kulmala et al., 2014). Therefore, it is possible that the cause of decreased plantar flexor power is gait disorder. According to a study by Suzuki, Bean & Fielding (2001), there is a correlation between muscle power of the plantar flexors and habitual or maximal gait speed in community-dwelling elderly women. However, limitations of this study are the small sample size and narrow age range. For the above reasons, we consider that preventative measures for the decline of walking ability include early detection and intervention of decreased plantar flexor power, and maintenance or improvement of walking speed.

Locomotive syndrome (LS) is defined as a decline in locomotor function as a result of locomotor organ impairment (Nakamura et al., 2015; Nakamura & Ogata, 2016). Healthy locomotor organs are essential for locomotor function, as well as for daily living and social activities. LS is bone and joint diseases such as osteoporosis-related fractures, spondylosis, and osteoarthritis of the knee joints associated with gait disorder (Nakamura, 2011). Previous studies reported that mobility dysfunction increases and ADL decline in LS, which increases the risk of requiring nursing care, the need for long-term care, and the risks of gait disorder and being bedridden (Ishibashi, 2018). In general, LS tests as risk assessments could be self-administered using Locomo25, which consists of 25 questions, a two-step test and the stand-up test (Muranaga & Hirano, 2003; Ogata et al., 2015).

The two-step test involves dividing the maximum stride length by the height of the patient, followed by the stand-up test, which involves standing on one or both legs at different heights. In particular, the two-step test performed in this study screens walking ability, and the stand-up test measures the muscle power of the lower extremity (Muranaga & Hirano, 2003; Ogata et al., 2015). Muranaga & Hirano (2003) reported that the two-step test can estimate walking ability, fall risk and independence level of ADL.

However these assessments demonstrate locomotion ability, they do not assess kinematics and kinetics or use multiple regression analysis that limits locomotor function using a three-dimensional motion analysis system such as a biomechanical method. Thus, we must draw attention to the cause and effect relationship with the two-step test and walking disability. For example, the two-step test is performed at the maximum stride length, thus the two-step test is assumed to be similar to the forward lunge. In previous studies using biomechanical analysis, the forward lunge was demonstrated to involve large hip, knee angle and hip extensor moments (Comfort et al., 2015; Riemann et al., 2013). Therefore, the two-step test is considered a strong reflection of hip joint function and knee joint function. Although LS has been reported to increase the risk of gait disorder, little attention has been given to the assessment of plantar flexor in the LS tests. Namely, it is evident that the cause of decreased walking speed is associated with a decrease in plantar flexor power during the terminal stance phase, but a conventional walking test does not include an evaluation of ankle joint function. For these reasons, we hypothesized that ankle joint function is more closely related to walking speed than hip joint function.

Gait is traditionally analyzed by undertaking a three-dimensional motion analysis (Winter et al., 1990; Judge, Ounpuu & Davis, 1996). This is regarded as the ‘golden standard’ method of measuring kinematics and kinetics. However the three-dimensional motion analysis systems are expensive, difficult to carry, and necessitates to measurements in the laboratory. Therefore, these are difficult to apply in clinical settings.

To overcome this problem, we developed a novel method for gait analysis using an inertial sensor to assess function of the ankle during the terminal stance phase of gait (Miyashita, Kudo & Maekawa, 2019). The validity of this method was verified using a three-dimensional motion analysis system. Differences of plantar flexor power measurements between from the three-dimensional motion analysis and inertial sensor were investigated using Bland–Altman analysis. It was found from the result that no systematic error in this methods using inertial sensor. Participants were equipped with a single inertial sensor mounted on the fibular head. We measured when vertical acceleration (Ay) peaked from heel-off (HO) to toe-off (TO) during the terminal stance phase. The estimated ankle power can be calculated from the acceleration (Ay) peak value obtained using the multiple regression equation (Miyashita, Kudo & Maekawa, 2019).

The purpose of this study was to investigate the relationship between walking speed and ankle power during the terminal stance of gait in community-dwelling middle-aged and elderly women using an inertial sensor.

## Materials and Methods

Participants aged over 50 to under 90 years were recruited from two orthopedic clinics and volunteers included community-dwelling healthy older individuals resident in urban and rural population centres in five prefectures (Osaka, Hyogo, Kyoto, Nara, Nagano) in Japan. The study duration was from April 2018 to October 2019. One hundred seventy-two healthy middle-aged to elderly community-dwelling women were included in this study. This study was approved by the Morinomiya University of Medical Science ethics committee (Approval number: 2018-009), and all subjects provided written informed consent.

**Figure 1:**
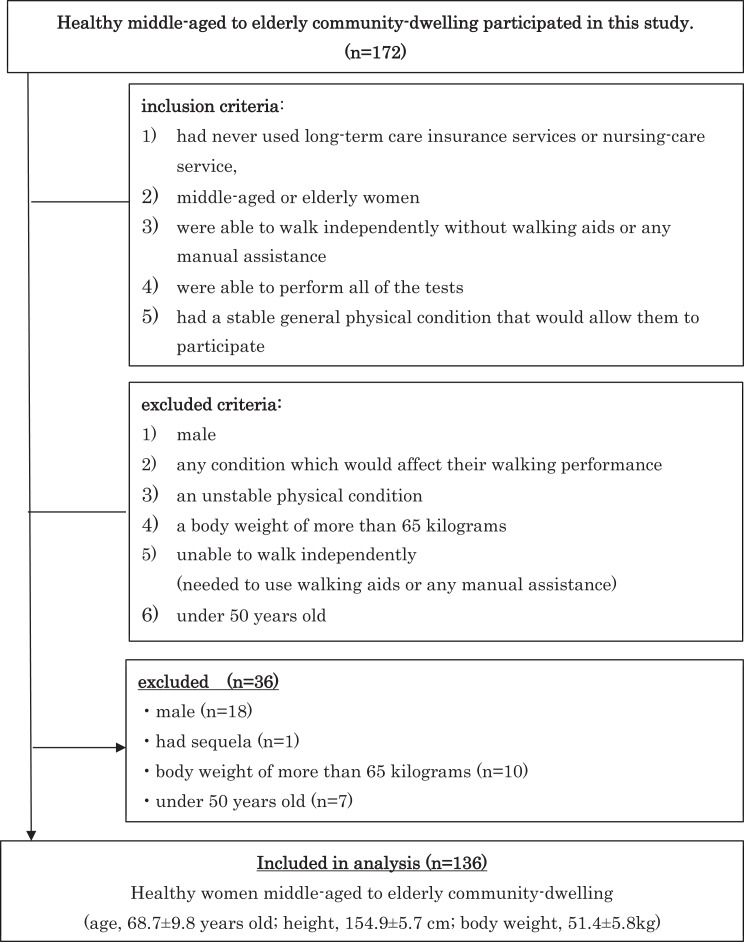
Flow chart of criteria in this study.

Participants were admitted to the study if they fulfilled the following inclusion criteria: (1) had never used long-term care insurance services or nursing-care service, (2) were middle-aged or elderly women, (3) were able to walk independently without walking aids or any manual assistance, (4) were able to perform all of the tests, (5) had a stable general physical condition that would allow them to participate in this study. Participants were excluded if they were: (1) male* (2) had any condition which would affect their walking performance †(3) had an unstable physical condition †(4) a body weight of more than 65 kg* (5) were unable to walk independently, that is, they needed to use walking aids or any manual assistance†, or (6) were under 50 years old. *Male and participants over 65 kg were excluded as they affect plantar flexor power in this study. †Also, items that affect the acceleration waveform were excluded during the walking. Insurance services were confirmed by interview medical history. Eighteen recruits were male, one recruit had sequelae affecting their walking, 10 had a body weight of more than 65 kg, and seven were under 50 years old. Finally, 136 healthy women participated in this study (Fig. 1).

Previous study reported the two-step test can evaluate walking ability (Muranaga & Hirano, 2003). The two-step value calculation involves dividing the maximum stride length of two steps by the height of the patient. The maximum stride length of the two-step test was measured using a measuring tape.

The grip strength is commonly and widely measured as an indicator of lower extremity muscle strength (Cooper et al., 2014). Grip strength was averaged over the values measured twice in the dominant hand using a Smedley type hand dynamometer (TOEI LIGHT Corporation T-1781).

**Figure 2:**
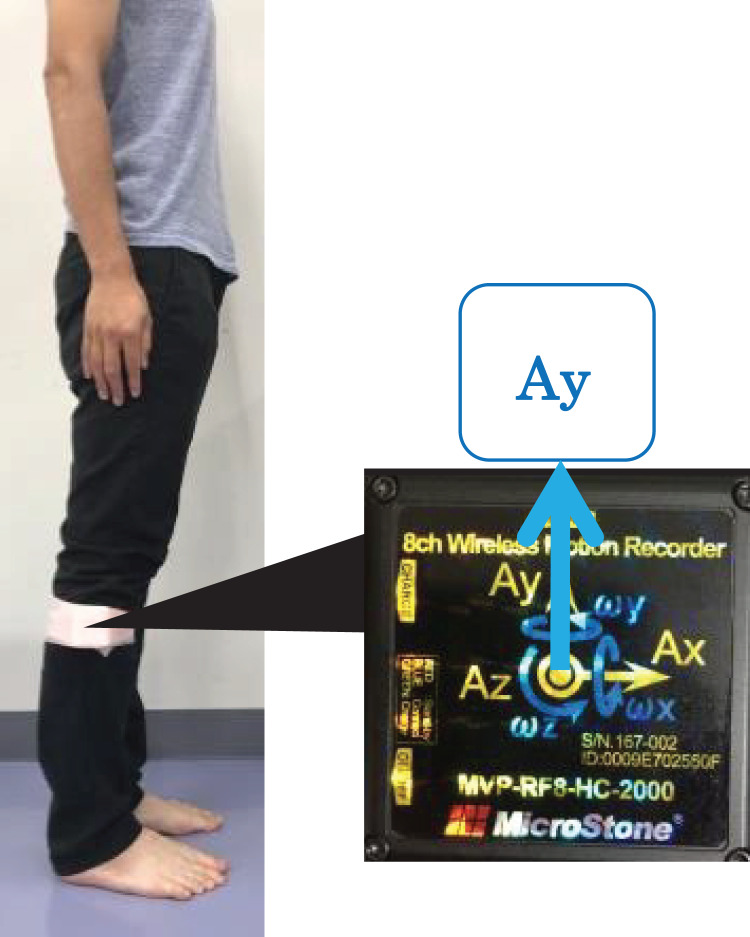
An inertial sensor was mounted on the lower leg at the fibula head.

In general, walking speed is useful to assess. Furthermore, walking speed is affected by age and muscle strength (Bohannon, 1997; Bohannon & Williams Andrews, 2011). Walking speed measurements were performed on a flat walkway with a total length of 9 m with road cones placed at both ends and with 2 m acceleration and deceleration distances. Each participant was instructed to walk at her own comfortable pace. All participants used everyday footwear. Comfortable walking speed measured using a stopwatch was averaged over the values measured twice. In addition, we measured estimated ankle power during gait using an inertial sensor (MicroStone Corporation, MVP-RF8-HC, sampling frequency, 100 Hz) mounted on the fibular head (Fig. 2). As a countermeasure against motion artifacts, the accelerometer was fixed with a Foam underwrap when it was mounted on the fibular head.

**Figure 3:**
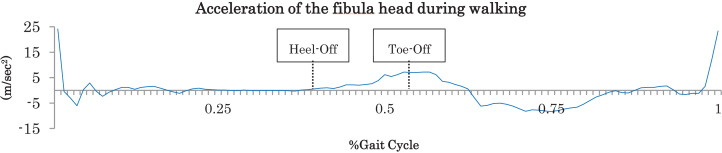
Acceleration of the fibula head during walking. The peak values of vertical direction from Heel-Off to Toe-Off were measured.

In our previous study (Miyashita, Kudo & Maekawa, 2019) using an inertial sensor we developed a method to calculate the estimated plantar flexors power from acceleration during the walking terminal stance. The target of analysis was the acceleration peak value from accelerometer data from which we estimated ankle power (estimated ankle power). The accelerometer data used raw data and not filtered. Acceleration peak value was measured on stable accelerometer waveform data during steady-state walking over approximately 13–14 steps (Fig. 3). The estimated ankle power was calculated using acceleration from an inertial sensor mounted on the fibular head during walking from HO to TO when a patient was walking at her own comfortable pace. The estimated ankle power was calculated as follows: estimated ankle power (W) = −4.689 + 0.269 × Ay + 0.104 × body weight. In addition, the estimated ankle power was normalized by body mass.

### Statistical analysis

All participants were classified into the four different age strata, fifties (50–59), sixties (60–69), seventies (70–79) and eighties (80–89). The differences in each parameter between the four age groups were compared using repeated analysis of variance (ANOVA), and post-hoc Bonferroni corrections for multiple comparisons to establish significance. Multiple regression analysis was carried out using a stepwise method to determine the correlations with comfortable walking speed. Comfortable walking speed was considered a dependent variable. Data of age, height, two-step value, grip strength and the normalized estimated ankle power items were considered independent variables. All statistical analyses were performed using IBM SPSS Statistics for Windows (IBM SPSS Statistics for Windows, Version 24.0. Armonk, NY, USA). Values of *P* < 0.05 were considered to indicate statistical significance for all tests. The sample size was estimated for a power of 0.8 and effect size of 0.33. The sample size was determined by GPower 3 Software (version 3.1.9.4) using the *F* test for one-way ANOVA. One hundred four subjects were deemed adequate, with 26 participants needed per group.

## Results

A total of 136 participants completed all assessments in this study. General characteristics, including two-step value, grip strength, comfortable walking speed, estimated ankle power and the normalized estimated ankle power of the participants, are summarized in Table 1.

**Table 1:** The association between age, height, body weight, two-step value, grip strength, walking speed, estimated ankle plantar flexor power and the normalized estimated ankle power of participants by decade year strata.

	Total (*n* = 136)	Fifties (50–59) (*n* = 28)	Sixties (60–69) (*n* = 42)	Seventies (70–79) (*n* = 40)	Eighties (80–89) (*n* = 26)	*P*	Effect size
Age (years)	68.7 (9.8)	54.5 (2.6)	64.9 (3.2)	73.8 (3.0)	82.1 (2.2)	50 < 60 s, 60 < 70 s, 70 < 80 s, 50 < 80 s	0.92
Height (cm)	154.9 (5.7)	159.4 (4.7)	154.8 (5.2)	154.7 (4.9)	150.8 (5.3)	80 < 70 s, 80 < 60 s, 80 < 50 s, 70 < 50 s, 60 < 50 s	0.22
Body weight (kg)	51.4 (5.8)	52.3 (5.9)	51.6 (4.5)	52.4 (7.0)	48.6 (4.7)	n.s	0.06
Two-step value	1.34 (0.16)	1.37 (0.15)	1.38 (0.15)	1.36 (0.12)	1.23 (0.19)	80 < 70 s, 80 < 60 s, 80 < 50 s	0.14
Grip strength (kg)	22.9 (4.7)	23.8 (5.0)	24.2 (5.1)	22.7 (4.5)	20.2 (2.7)	80 < 60 s, 80 < 50 s	0.10
Walking speed (m/s)	1.37 (0.24)	1.44 (0.26)	1.37 (0.26)	1.39 (0.19)	1.23 (0.23)	80 < 70 s, 80 < 50 s	0.10
Estimated ankle power (W)	3.12 (0.85)	3.40 (0.88)	3.12 (0.79)	3.28 (0.86)	2.57 (0.68)	80 < 70 s, 80 < 60 s, 80 < 50 s	0.11
Normalized estimated Ankle power	0.060 (0.014)	0.065 (0.014)	0.060 (0.014)	0.062 (0.011)	0.053 (0.013)	80 < 70 s, 80 < 50 s	0.08

**Note:**

ANOVA (*P* < 0.05), post-hoc Bonferroni corrections 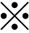
ave (SD).

Results were compared among the four different age strata, fifties to eighties. The estimated ankle power of the eighties group was significantly decreased in comparison with other age groups (*P* < 0.05), but there were no significant differences in estimated ankle power among the fifties to seventies age-groups (Table 1). Similarly, the normalized estimated ankle power of the eighties group was significantly decreased in comparison with seventies age groups and fifties groups (*P* < 0.05) (Table 1). Meanwhile, there were no significant differences in normalized estimated ankle power between the sixties and eighties age-groups.

The results of stepwise multiple regression analysis are shown in Table 2. The analysis revealed that the normalized estimated ankle power, two-step value and body weight were highly-significant partial regression coefficients (adjusted *R*^2^ = 0.57, Table 2).

**Table 2:** Results of multiple regression analysis (dependent variable: walking speed).

	Unstandardized coefficients	Standardized coefficients β	95% Confidence interval		*P*-value	VIF
			Lower bound	Upper bound		
(Constant)	0.175		−0.153	0.502	<0.05	
Estimated Ankle plantar flexion power	9.425	0.532	7.263	11.586	<0.01	1.187
Two-step score	0.653	0.425	0.472	0.833	<0.01	1.102
Body Weight	−0.005	−0.119	−0.010	−0.000	<0.05	1.081

**Note:**

Adjusted *R*^2^ = 0.57.

## Discussion

The purpose of this study was to reveal the relationship between walking speed and plantar flexor power during the terminal stance of gait in community-dwelling middle-aged and elderly women using an inertial sensor. We hypothesized that estimated ankle power decreases with age, and that estimated ankle power is more closely related to walking speed than two-step value. The grip strength and the walking speed of participants in their eighties in this study were not below the cut-off values of frailty or sarcopenia screening tests (Lee et al., 2017; Walston, Buta & Xue, 2018; Chen, Lee & Peng, 2016). Therefore, the participants of this study were identified as robust elderly among the community-dwelling elderly. The most influential independent determinant of walking speed was found to be the normalized estimated ankle power (β = 0.532) in the stepwise regression analysis. Furthermore, we also found that two-step score (β = 0.425), and body weight (β = −0.119) were related. As expected, the normalized estimated ankle power was most related to comfortable walking speed. Regarding the relationship with age, the normalized estimated ankle power generally decreased with age. The normalized estimated ankle power and walking speed in their eighties were significantly decreased than both that of fifties and seventies. However, there was no significant difference in normalized estimated ankle power and walking speed between the sixties and eighties.

It is known that the plantar flexor moment and the plantar flexors power activities have large affects through the mid-stance to pre-swing in normal walking (Perry & Burnfield, 2010). Moreover, in the sagittal plane during walking, plantar flexor power plays a role in acceleration to propel the body forward during the terminal stance (Neptune, Kautz & Zajac, 2001; Neptune & McGowan, 2011; Liu et al., 2006). Several previous studies using a three-dimensional motion analysis system clearly showed that the main factor in the decrease of walking speed with aging was the decline of plantar flexor power (Winter et al., 1990; Judge, Ounpuu & Davis, 1996; Franz & Kram, 2013; Kulmala et al., 2014). Most of the older adult participants included in previous studies were in their seventies, and the sample size was less than twenty participants. In addition, the older adults were compared to young adult participants as a control group; there were no comparisons to those nearer their age range. Therefore, it was not sufficient to consider changes with aging, such as whether the plantar flexor power of subjects in their seventies was lower than those of participants in their thirties to fifties. We analyzed plantar flexor power using an inertial sensor which is typically lightweight, portable, and easy to use, and we were able to gather data from many middle-aged and elderly participants. As a result, we found that normalized estimated ankle power and comfortable walking speed were maintained into the fifties and seventies. In addition, it was revealed that the normalized estimated ankle power was significantly decreased in robust elderly people when they entered their eighties compare to the fifties and seventies.

When compared, the estimated ankle power calculated by the inertial sensor used in this study and the value of the plantar flexor power of participants in their seventies calculated by three-dimensional motion analysis in previous studies, the average value of this study was 3.28 W and standard deviation 0.86, whereas Graf et al. (2005) obtained a value of 3.00 W and standard deviation 0.88, for Kulmala et al. (2014) it was 3.00 W standard deviation 0.8, and in the study by Browne & Franz (2018) the value was 2.79 W and standard deviation 0.66. As can be seen above, in each case the average value obtained from previous studies was within the range of the average value ±1 SD of this study, supporting the validity of the estimated ankle power calculated by the inertial sensor in this study.

In this study we found that the normalized estimated ankle power of robust elderly people in their eighties was significantly lower than that of those aged in their fifties and seventies. In the biomechanics of human walking, it is clear that ankle joint function decreases with aging. Also, the mechanical work expenditure of the ankle in the sagittal plane of comfortable walking begins to decrease from middle age (Ko et al., 2012). A recent study reported that walking speed affected ankle power (Da Silva et al., 2020). It was unexpected to observe that the normalized estimated power of the sixties group was not significantly greater than the eighties group. Although not significant, the sixties group also had a lower normalized estimated power output than the fifties and seventies. We hypothesize that this may have been due to there being a trade-off between ankle function and function at the hip or knee in the sixties group. When ankle function is reduced, the other joints in the lower limb will compensate (Lewis & Ferris, 2008; Judge, Davis & Ounpuu, 1996). A measure of hip and knee function is the two-step test. Although not significant, the two-step values of the sixties group were the largest of all groups which may suggest that the hip and knee were compensating for reduced ankle joint function in this group. This may explain why the sixties group had a lower walking speed and normalized estimated ankle power than the fifties and seventies, and why their values were not significantly lower than the eighties group. Therefore, because the reduced in the normalized estimated ankle power value was compensated for by the hip joint function, it was considered that the walking speed in the sixties was faster than in the eighties. And, the normalized estimated ankle power value in the sixties was not significantly different from that in the eighties. Moreover, although the estimated ankle power of the eighties group was significantly decreased, the effect size was small-trivial. Therefore, the difference in ankle power output is not large. Although walking speed was maintained, estimated ankle power was observed to undergo a reduction in older age-group participants, and there were also cases where the estimated ankle power fell below the average value for the older ages. Namely, regarding the seventies age-group participants, although twenty-two people had a walking speed above average, three of the participants had ankle power lower than the average in the eighties age-group.

In previous studies it was necessary to obtain a three-dimensional motion analysis system to perform kinetic analysis. However, we have shown that it is possible to easily quantify plantar flexor power using an inertial sensor during walking in order to detect a walking disorder. There is therefore a possibility of detecting a decline in walking ability early as LS with age-related changes. This finding is likely to have significance for clinical treatment.

There are some limitations to this study. First, our method using an inertial sensor can measure walking outdoors, but this method is dependent on the external environment such as the road surface and the road slope. Therefore, it was necessary to use a flat walkway in this study. Second, the analysis of estimated ankle power is difficult because the unclear accelerometer waveform of HO–TO may not provide accurate lower limb acceleration signals during walking. Third, it is necessary to increase male participants. Fourth, we hypothesized that both the normalized estimated ankle power and walking speed in the eighties were significant lower than those of the sixties groups. However, those parameters were not significantly different between the sixties and eighties group, although those of the sixties group showed tendency to be greater values. In this study, it is possible that residential areas of participants or regular physical activity such as exercise habits may influence on walking abilities. Further study, it is needed to be clarified it in prospective cohort study. In addition, in order to clarify the relationship between plantar flexor power and walking speed, it will be necessary to examine individuals with greater body mass and include males and perform an intervention study of the plantar flexor power. Finally, it will be necessary to control walking speed when investigating the age-related effects of ankle power.

## Conclusions

In conclusion, the ankle joint power during walking is suggested to be highly related to walking speed. It is considered important not only to use the walking speed, but also to evaluate plantar flexor power. We suggest that ankle function should be assessed in combination with hip function when assessment of locomotor function is performed to evaluate walking and enable early detection of disability. Our method will be useful in allowing for early prophylaxis of walking disability in community-dwelling middle-aged and elderly people with reduced plantar flexor power during walking.

## Supplemental Information

10.7717/peerj.11269/supp-1 Supplemental Information 1 Data of age, height, weight, two-step value, grip strength, comfortable walking speed, the estimated ankle power and the normalized estimated ankle power items of all the participants.

10.7717/peerj.11269/supp-2 Supplemental Information 2 Image accelerometer.

10.7717/peerj.11269/supp-3 Supplemental Information 3 An inertial sensor mounted on the lower leg at the fibula head. As a countermeasure against motion artifacts, the inertial sensor was fixed with a Foam underwrap when it was mounted on the fibular head.
During the terminal stance phase, the vertical acceleration (Ay) was measured by the inertial sensor mounted on the fibular head.

## Additional Information and Declarations

### Data Availability

The following information was supplied regarding data availability: The raw measurements are available in the Supplemental File.

## References

[ref-2] Bohannon RW (1997). Comfortable and maximum walking speed of adults aged 20–79 years: reference values and determinants. Age and Ageing.

[ref-3] Bohannon RW, Williams Andrews A (2011). Normal walking speed: a descriptive meta-analysis. Physiotherapy.

[ref-4] Browne MG, Franz JR (2018). More push from your push-off: joint-level modifications to modulate propulsive forces in old age. PLOS ONE.

[ref-5] Chen L, Lee W, Peng L (2016). Recent advances in sarcopenia research in Asia: 2016 update from the Asian working group for sarcopenia. Journal of the American Medical Directors Association.

[ref-6] Comfort P, Jones PA, Smith LC, Herrington L (2015). Joint kinetics and kinematics during common lower limb rehabilitation exercises. Journal of Athletic Training.

[ref-7] Cooper C, Fielding R, Visser M, Van Loon LJ, Rolland Y, Orwoll E, Reid K, Boonen S, Dere W, Epstein S, Mitlak B, Tsouderos Y, Sayer AA, Rizzoli R, Reginster JY, Kanis JA (2014). Tools in the assessment of sarcopenia. Calcified Tissue International.

[ref-8] Cummings SR, Studenski S, Ferrucci L (2014). A diagnosis of dismobility—giving mobility clinical visibility: a mobility working group recommendation. JAMA.

[ref-9] Da Silva LS, Fukuchi RK, Watanabe RN, Fukuchi CA, Duarte M (2020). Effects of age and speed on the ankle-foot system’s power during walking. Scientific Reports.

[ref-10] Franz JR, Kram R (2013). Advanced age affects the individual leg mechanics of level, uphill, and downhill walking. Journal of Biomechanics.

[ref-11] Fritz S, Lusardi M (2009). White paper: “walking speed: the sixth vital sign”. Journal of Geriatric Physical Therapy.

[ref-12] Fulk GD, He Y, Boyne P, Dunning K (2017). Predicting home and community walking activity poststroke. Stroke.

[ref-13] Graf A, Judge JO, Õunpuu S, Thelen DG (2005). The effect of walking speed on lower-extremity joint powers among elderly adults who exhibit low physical performance. Archives of Physical Medicine and Rehabilitation.

[ref-14] Hollman JH, McDade EM, Petersen RC (2011). Normative spatiotemporal gait parameters in older adults. Gait & posture.

[ref-15] Ishibashi H (2018). Locomotive syndrome in Japan. Osteoporosis and Sarcopenia.

[ref-16] Judge JO, Davis RB, Ounpuu S (1996). Step length reductions in advanced age the role of ankle and hip kinetics. Journals of Gerontology Series A: Biological Sciences and Medical Sciences.

[ref-17] Judge JO, Ounpuu S, Davis RB (1996). Effects of age on the biomechanics and physiology of gait. Clinics in Geriatric Medicine.

[ref-18] Ko S, Stenholm S, Metter EJ, Ferrucci L (2012). Age-associated gait patterns and the role of lower extremity strength—results from the Baltimore Longitudinal Study of Aging. Archives of Gerontology and Geriatrics.

[ref-19] Kulmala J-P, Korhonen MT, Kuitunen S, Suominen H, Heinonen A, Mikkola A, Avela J (2014). Which muscles compromise human locomotor performance with age?. Journal of the Royal Society, Interface.

[ref-20] Lee L, Fm M, Coe C, Tejal F, Pharmd P, Costa A, Bryce E, Hillier LM (2017). Screening for frailty in primary care Recherche Dépister les patients fragiles dans un contexte de soins primaires. Canadian Family Physician.

[ref-21] Lewis CL, Ferris DP (2008). Walking with increased ankle pushoff decreases hip muscle moments. Journal of Biomechanics.

[ref-22] Liu MQ, Anderson FC, Pandy MG, Delp SL (2006). Muscles that support the body also modulate forward progression during walking. Journal of Biomechanics.

[ref-23] May D, Nayak US, Isaacs B (1985). The life-space diary: a measure of mobility in old people at home. International Rehabilitation Medicine.

[ref-24] Meinders M, Gitter A, Czerniecki JM (1998). The role of ankle plantar flexor muscle work during walking. Scandinavian Journal of Rehabilitation Medicine.

[ref-25] Menz HB, Lord SR, Fitzpatrick RC (2003a). Age-related differences in walking stability. Age and Ageing.

[ref-26] Menz HB, Lord SR, Fitzpatrick RC (2003b). Acceleration patterns of the head and pelvis when walking are associated with risk of falling in community-dwelling older people. Journals of Gerontology Series A: Biological Sciences and Medical Sciences.

[ref-27] Miyashita T, Kudo S, Maekawa Y (2019). Estimation of the ankle power during the terminal stance of gait using an inertial sensor. Journal of Physical Therapy Science.

[ref-28] Muranaga S, Hirano K (2003). Development of a convenient way to predict ability to walk using a two-step test. Journal of the Showa Medical Association.

[ref-29] Nakamura K (2011). The concept and treatment of locomotive syndrome: its acceptance and spread in Japan. Journal of Orthopaedic Science.

[ref-30] Nakamura K, Ogata T (2016). The concept and treatment of locomotive syndrome: its acceptance and spread in Japan. Clinical Reviews in Bone and Mineral Metabolism.

[ref-31] Nakamura K, Yoshimura N, Ogata T, Akune T, Tobimatsu Y (2015). The concept of locomotive syndrome and its relationship with frailty and sarcopenia. Nihon Rinsho—Japanese Journal of Clinical Medicine.

[ref-32] Neptune RR, Kautz SA, Zajac FE (2001). Contributions of the individual ankle plantar flexors to support, forward progression and swing initiation during walking. Journal of Biomechanics.

[ref-33] Neptune RR, McGowan CP (2011). Muscle contributions to whole-body sagittal plane angular momentum during walking. Journal of Biomechanics.

[ref-34] Ogata T, Muranaga S, Ishibashi H, Ohe T, Izumida R, Yoshimura N, Iwaya T, Nakamura K (2015). Development of a screening program to assess motor function in the adult population: a cross-sectional observational study. Journal of Orthopaedic Science: Official Journal of the Japanese Orthopaedic Association.

[ref-35] Perry J, Burnfield JM (2010). Gait analysis: normal and pathological function.

[ref-36] Perry J, Garrett M, Gronley JK, Mulroy SJ (1995). Classification of walking handicap in the stroke population. Stroke.

[ref-37] Päivi M, Mirja H, Terttu P (2010). Changes in physical activity involvement and attitude to physical activity in a 16-year follow-up study among the elderly. Journal of Aging Research.

[ref-38] Rantakokko M, Portegijs E, Viljanen A, Iwarsson S, Rantanen T (2013). Life-space mobility and quality of life in community-dwelling older people. Journal of the American Geriatrics Society.

[ref-39] Riemann B, Congleton A, Ward R, Davies GJ (2013). Biomechanical comparison of forward and lateral lunges at varying step lengths. Journal of Sports Medicine and Physical Fitness.

[ref-40] Schmid A, Duncan PW, Studenski S, Lai SM, Richards L, Perera S, Wu SS (2007). Improvements in speed-based gait classifications are meaningful. Stroke.

[ref-41] Simpson ME, Serdula M, Galuska DA, Gillespie C, Donehoo R, Macera C, Mack K (2003). Walking trends among U.S. adults: the behavioral risk factor surveillance system, 1987–2000. American Journal of Preventive Medicine.

[ref-42] Studenski S, Perera S, Patel K, Rosano C, Faulkner K, Inzitari M, Brach J, Chandler J, Cawthon P, Connor EB, Nevitt M, Visser M, Kritchevsky S, Badinelli S, Harris T, Newman AB, Cauley J, Ferrucci L, Guralnik J (2011). Gait speed and survival in older adults. JAMA.

[ref-43] Sustakoski A, Perera S, VanSwearingen JM, Studenski SA, Brach JS (2015). The impact of testing protocol on recorded gait speed. Gait & Posture.

[ref-44] Sutherland DH, Cooper L, Daniel D (1980). The role of the ankle plantar flexors in normal walking. Journal of Bone and Joint Surgery.

[ref-45] Suzuki T, Bean JF, Fielding RA (2001). Muscle power of the ankle flexors predicts functional performance in community-dwelling older women. Journal of the American Geriatrics Society.

[ref-46] Tudor-Locke C, Craig CL, Aoyagi Y, Bell RC, Croteau KA, De Bourdeaudhuij I, Ewald B, Gardner AW, Hatano Y, Lutes LD, Matsudo SM, Ramirez-Marrero FA, Rogers LQ, Rowe DA, Schmidt MD, Tully MA, Blair SN (2011). How many steps/day are enough? For older adults and special populations. International Journal of Behavioral Nutrition and Physical Activity.

[ref-47] Verlinden VJA, Van der Geest JN, Hoogendam YY, Hofman A, Breteler MMB, Ikram MA (2013). Gait patterns in a community-dwelling population aged 50 years and older. Gait & posture.

[ref-48] Viosca E, Martinez JL, Almagro PL, Gracia A, Gonzalez C (2005). Proposal and validation of a new functional ambulation classification scale for clinical use. Archives of Physical Medicine and Rehabilitation.

[ref-49] Walston J, Buta B, Xue QL (2018). Frailty screening and interventions: considerations for clinical practice. Clinics in Geriatric Medicine.

[ref-50] Winter DA, Patla AE, Frank JS, Walt SE (1990). Biomechanical walking pattern changes in the fit and healthy elderly. Physical Therapy.

[ref-51] Yoshimura N, Muraki S, Oka H, Tanaka S, Ogata T, Kawaguchi H, Akune T, Nakamura K (2015). Association between new indices in the locomotive syndrome risk test and decline in mobility: third survey of the ROAD study. Journal of Orthopaedic Science: Official Journal of the Japanese Orthopaedic Association.

