# Supplementary figures and images for: Assessment of walking disorder in community-dwelling Japanese middle-aged and elderly women using an inertial sensor
Source: PeerJ. 2021 Apr 14;9:e11269. doi: 10.7717/peerj.11269 (PMC8052961; doi:10.7717/peerj.11269)

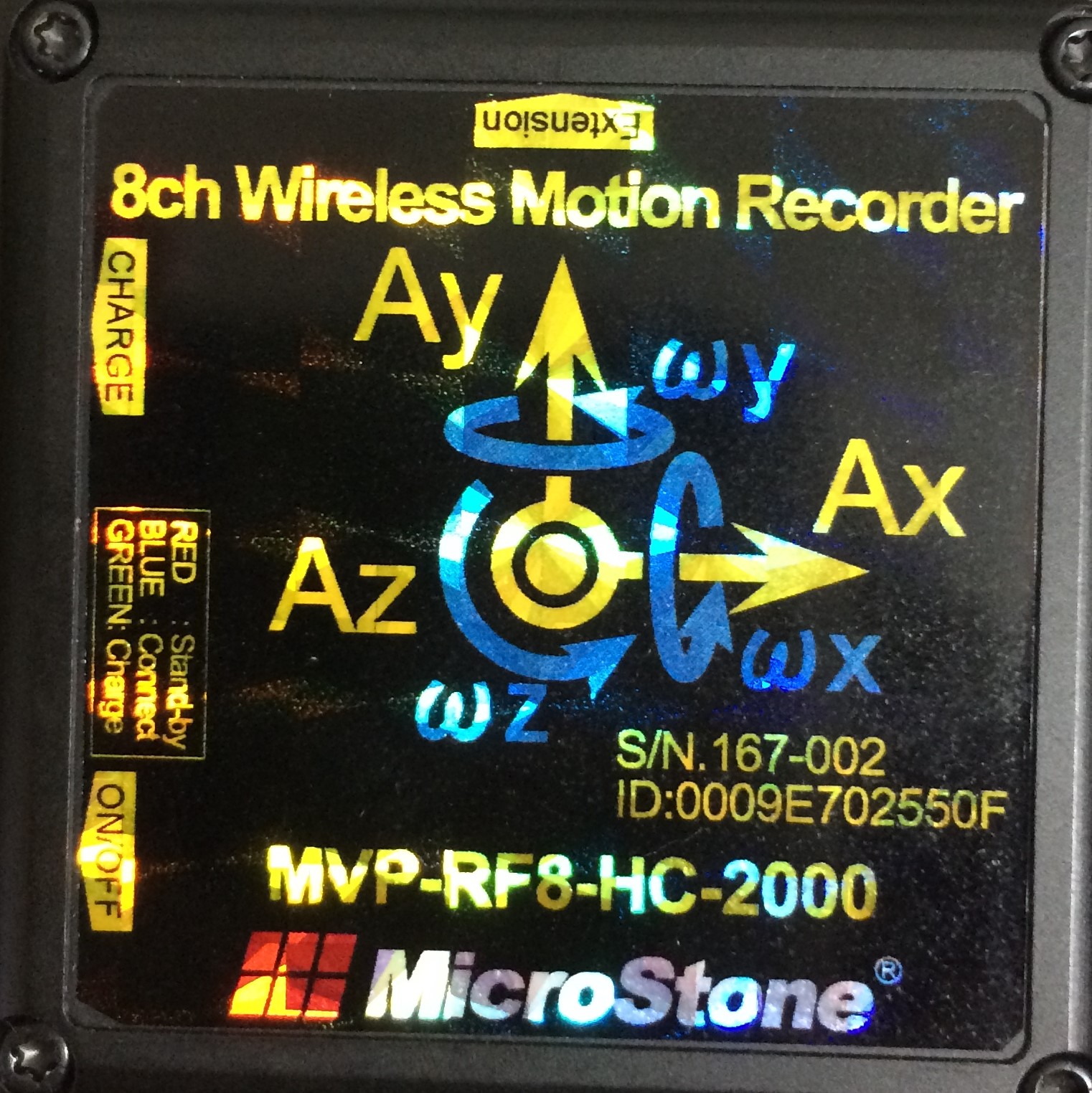

Supplement: Supplemental Information 2 [file peerj-09-11269-s002.jpg]

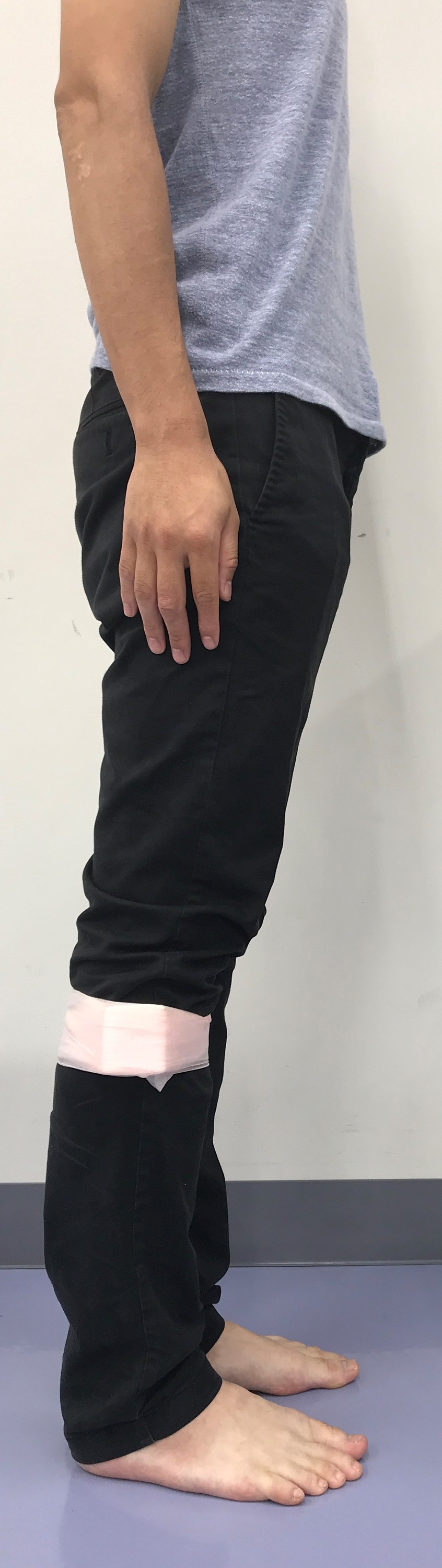

Supplement: Supplemental Information 3 — As a countermeasure against motion artifacts, the inertial sensor was fixed with a Foam underwrap when it was mounted on the fibular head. During the terminal stance phase, the vertical acceleration (Ay) was measured by the inertial sensor mounted on the fibular head. [file peerj-09-11269-s003.jpg]
